# A Novel High-Throughput Approach to Measure Hydroxyl Radicals Induced by Airborne Particulate Matter

**DOI:** 10.3390/ijerph121113678

**Published:** 2015-10-28

**Authors:** Yeongkwon Son, Vladimir Mishin, William Welsh, Shou-En Lu, Jeffrey D. Laskin, Howard Kipen, Qingyu Meng

**Affiliations:** 1 Department of Environmental and Occupational Health, School of Public Health, Rutgers University, Piscataway, NJ 08854, USA; E-Mails: sonye@sph.rutgers.edu (Y.S.); jlaskin@eohsi.rutgers.edu (J.D.L.); kipen@eohsi.rutgers.edu (H.K.); 2 Department of Pharmacology and Toxicology, Pharmacy College, Rutgers University, Piscataway, NJ 08854, USA; E-Mail: mishin@eohsi.rutgers.edu; 3 Environmental and Occupational Health Sciences Institute, Rutgers University, Piscataway, NJ 08854, USA; E-Mails: welshwj@rwjms.rutgers.edu (W.W.); shouen.lu@rutgers.edu (S.-E.L.); 4 Department of Pharmacology, Robert Wood Johnson Medical School, Rutgers University, Piscataway, NJ 08854, USA; 5 Department of Biostatistics, School of Public Health, Rutgers University, Piscataway, NJ 08854, USA

**Keywords:** hydroxyl radical, oxidative potential, oxidative stress, particulate matter, air pollution, high throughput analysis, molecular probe, exposure assessment

## Abstract

Oxidative stress is one of the key mechanisms linking ambient particulate matter (PM) exposure with various adverse health effects. The oxidative potential of PM has been used to characterize the ability of PM induced oxidative stress. Hydroxyl radical (•OH) is the most destructive radical produced by PM. However, there is currently no high-throughput approach which can rapidly measure PM-induced •OH for a large number of samples with an automated system. This study evaluated four existing molecular probes (disodium terephthalate, 3′-*p*-(aminophenyl)fluorescein, coumarin-3-carboxylic acid, and sodium benzoate) for their applicability to measure •OH induced by PM in a high-throughput cell-free system using fluorescence techniques, based on both our experiments and on an assessment of the physicochemical properties of the probes reported in the literature. Disodium terephthalate (TPT) was the most applicable molecular probe to measure •OH induced by PM, due to its high solubility, high stability of the corresponding fluorescent product (*i.e.*, 2-hydroxyterephthalic acid), high yield compared with the other molecular probes, and stable fluorescence intensity in a wide range of pH environments. TPT was applied in a high-throughput format to measure PM (NIST 1648a)-induced •OH, in phosphate buffered saline. The formed fluorescent product was measured at designated time points up to 2 h. The fluorescent product of TPT had a detection limit of 17.59 nM. The soluble fraction of PM contributed approximately 76.9% of the •OH induced by total PM, and the soluble metal ions of PM contributed 57.4% of the overall •OH formation. This study provides a promising cost-effective high-throughput method to measure •OH induced by PM on a routine basis.

## 1. Introduction

Exposure to ambient PM has been associated with a suite of adverse health effects, including cardiopulmonary diseases, metabolic diseases, and adverse birth outcomes [1,2,3,4]. Oxidative stress has been repeatedly reported as one of the underlying mechanisms linking PM exposures and adverse health outcomes [1,5,6,7,8,9,10].

Oxidative potential, which measures the capability of PM to induce oxidative stress [10], has been associated with cytotoxicity and adverse human health outcomes [3,11,12,13,14]. For example, the *in vitro* toxicity of RAW 264.7 cells has been associated with PM oxidative potential, measured as dithiothreitol (DTT) depletion [15], during the European RAPTES project [14]. In a panel epidemiologic study, Delfino *et al.* [11] also reported an association between DTT depletion induced by PM_2.5_ and the increase in FeNO. In another study, both DNA damage in A549 cells and the formation of 8-hydroxydeoxyguanosine in calf thymus DNA have been associated with the ability of PM to induce OH radicals [13]. In addition, PM oxidative potential has demonstrated significant spatial variations, and no routinely measured PM species, such as PM mass and elemental analysis, could sufficiently explain the spatial variations in oxidative potential [16], indicating the necessity to measure oxidative potential on a routine basis.

Most of the existing oxidative potential measurement methods target nonspecific (or non-identified) reactive oxygen species (ROS) and suffer from major analytical challenges [17,18], although each approach has its advantages and limitations (see Table S1 in the Supplementary Information for details). The commonly used DCFH-DA assay, *i.e.*, the oxidation of 2,7-dichlorodihydrofluorescein, was initially designed to measure hydrogen peroxide, but it was found to be oxidized by other ROS too, and suffered from auto-oxidation of dichlorofluorescein (DCF), high background signals, and the lack of robustness [17]. Neither the DTT depletion assay nor the antioxidant (e.g., ascorbic acid) depletion assays could assess specific ROS formation [15,19,20,21]. In addition, catalase, which catalyzes the removal of hydrogen peroxide, does not affect DTT assay results, indicating that the DTT assay missed radicals formed through the Fenton reaction, which is a major mechanism for the formation of ROS [18]. It is also worth noting that existing ROS measurement methods might not be directly comparable due to the lack of specificity of target ROS detection.

With the recognition of ROS-specific oxidative potential measurements being important to understand the linkage between oxidative potential and oxidative stress [17], methods have been developed to measure specific ROS, especially targeting the hydroxyl radical or •OH. Hydroxyl radical is the most reactive and destructive radical, and can attack all biological molecules, causing lipid peroxidation and DNA damage [7,10,13,22]. A few studies assessed PM-induced •OH formation using (1) trapping •OH with 5,5-dimethyl-1-pyrroline N-oxide followed by electron spin resonance analysis, and (2) capturing •OH with sodium benzoate followed by high performance liquid chromatography and ultraviolet/fluorescence detection [23,24,25,26,27]. However, these •OH measurement methods are labor- and time-intensive, and their use in routine analysis has thus been constrained [28,29,30]. Therefore, a method compatible with high-throughput techniques capable of measuring specific ROS is needed to measure •OH induced by PM.

Indeed, molecular probes, which react with •OH and form fluorescent products for high-throughput detection, exist for •OH measurements. The molecular probes used in previous studies include disodium terephthalate (TPT) [31,32], coumarin-3-carboxylic acid (3CCA) [33,34], 3′-*p*-(aminophenyl)-fluorescein (APF) [24,35] and benzoic acid (BA) [36,37]. However, these molecular probes were only applied in material sciences or biochemistry [32,34,35,37,38], and they have not been validated in air quality studies. It is largely unknown whether these fluorescence probes are suitable for PM-induced •OH measurements, under the reaction and measurement conditions different from previous studies with respect to pH levels and sensitivity.

In this study, four fluorescence probes (*i.e.*, TPT, APF, 3CCA and BA) were assessed for their potential capability to measure •OH induced by PM in a high-throughput approach. Fluorescence probes were assessed in terms of their sensitivity, reactivity, and specificity, based on experiments and the review of their physicochemical properties from existing literature.

## 2. Experimental Section

### 2.1. Molecular Probes and Reaction Products Detection

Four molecular probes were evaluated in this study: disodium terephthalate (TPT), 3′-*p*-(aminophenyl) fluorescein (APF), coumarin-3-carboxylic acid (3CCA), and sodium benzoate (BA). These molecular probes react with •OH and form final products for detection. The corresponding chemicals used for detection and quantifying •OH formation were 2-hydroxyterephthalic acid (2OHTA), fluorescein sodium salt (FL), 7-hydroxycoumarin-3-carboxylic acid (7OHCCA), and sodium salicylate (2OHBA), respectively. The purities and manufacturers of chemicals used in this study are presented in the Supplementary Information (Table S2).

A fluorescence approach was applied to quantify 2OHTA, FL, 7OHCCA, and 2OHBA, in UV transparent 96-well microplates (UV-Star^®^ 96-Well Microplates, Greiner Bio-One North America Inc., Monroe, NC, USA), using a microplate reader (SpectraMax M5, Molecular Devices, Sunnyvale, CA, USA) with bottom reading. The excitation/emission wavelengths were set based on previous publications, at 310/425 nm for 2OHTA [39], 492/525 nm for FL [35], 395/450 nm for 7OHCCA [34], and 300/410 nm for 2OHBA [36], respectively.

### 2.2. Molecular Probe Evaluation

Each molecular probe was evaluated for the following aspects: (1) sensitivity, measured as the limit of detection; (2) the rate constant of the reaction between a molecular probe and •OH; (3) the reaction yield, which is the amount of a product obtained in a chemical reaction; (4) the stability of a fluorescence product, *i.e.*, further reaction between a product and •OH and the depletion of fluorescence intensity; (5) the solubility of a molecular probe; and (6) the impact of pH on a product’s fluorescence intensity. In our study, the first and partially the second and third aspects were evaluated through experiments; and the rest of the aspects were evaluated through assessing existing literature on the physicochemical properties of molecular probes and the corresponding products.

### 2.3. PM-Induced Hydroxyl Radical Formation

#### 2.3.1. Reaction Environment

Hydroxyl radicals were induced by PM and its constituents under physiologically relevant conditions: at 37 °C, avoiding light, and under pH 7.4 [40,41]. The acidity of the solution was maintained using phosphate buffered saline (PBS), containing 114 mM sodium chloride, 8.0 mM Na_2_HPO_4_ and 2.0 mM KH_2_PO_4_ [18,40,41,42,43]. In addition, 100 μM of ascorbic acid was added into the buffer solution. Ascorbic acid is a natural component in human lung epithelial lining fluid, which serves as the first line of defense against exogenous substances as an antioxidant [41]. When ascorbic acid encounters PM, it forms ROS, including •OH, and ascorbic acid itself is depleted. The depletion of ascorbic acid has been used as a metric to measure PM oxidative potential in previous studies [10,21], although the amount of •OH formed from this process is unknown. The concentration of ascorbic acid used in this study (100 μM) is based on the concentration of ascorbic acid in human lung epithelial lining fluid [40,44,45]. All deionized water used in this study was treated with a Chelex-100 resin column to remove the potential contamination with metal ions [46].

#### 2.3.2. Hydroxyl Radical Induced by Transition Metal Ions

Initially, molecular probes were tested and applied to measure •OH induced by two transition metal ions found in ambient PM, Cu^2+^ and Fe^3+^ (High-Purity Standards, Charleston, SC, USA), through Fenton or Fenton-like reactions [23,47]. Molecular probes were first added to the buffer solution mentioned above, containing 100 μM of ascorbic acid in PBS, and then the reaction initiated by adding 100 μM of Cu^2+^ or Fe^3+^. The final volume of the reaction system was 200 μL and the reaction was conducted in a UV transparent 96-well microplate within the microplate reader, maintaining at 37 °C and avoiding light. The formation of 2OHTA, FL, 7OHCCA, or 2OHBA was measured every 15 min over a 2-h period, and 2OHTA, FL, 7OHCCA, or 2OHBA was quantified using calibration curves.

The saturated concentration of molecular probes was determined by examining the change in fluorescence intensity with increasing concentrations of molecular probes, *i.e.*, 0.05–20 mM of TPT, 1–125 μM of APF, 0.05–15 mM of 3CCA, and 0.05–20 mM of BA. When the measured fluorescence intensity reached a plateau, it indicated that the corresponding amount of molecular probe would be high enough and not a limiting factor in the assay (see Figure S1 in the Supplementary Information).

The concentration of Cu^2+^ or Fe^3+^ used in the experiment was based on their ambient air concentrations (Mean and median was 5.0 and 2.5 ng/m^3^ for Cu, and 98.4 and 63.1 ng/m^3^ for Fe) in the US (see Table S3, and the U.S. EPA Air Quality System [48]) and based on the following dosimetry assumptions: (1) a person inhales 20 m^3^ of air per day [49]; (2) 20% of inhaled particles deposit in the lung [50,51]; (3) the volume of epithelial lining fluid is 25 mL for a typical person [52,53]; and (4) particle deposition in the lung is not evenly distributed, as demonstrated by dosimetry models [50,51,54,55], which suggest that 100 times more particles are deposited at hotspots than other places in human lungs.

A control for dependence of fluorescence changes on •OH formation was applied under all experimental conditions by adding 50 mM of dimethyl sulfoxide (DMSO) into the system to quench the formed •OH.

#### 2.3.3. Hydroxyl Radicals Induced by PM

Hydroxyl radicals induced by PM were tested using a PM standard reference material from the National Institute of Standards and Technology (NIST SRM 1648a, urban dust). The •OH induction capability was tested with the total suspended PM, the soluble fraction of PM, and the fraction of PM with metals removed.

The PM suspension was prepared by sonication of PM for 5 min in PBS with 55 W power in water bath at room temperature. The chosen sonication time was sufficient to elute most of soluble fraction of PM (see Figure S2 in the Supplementary Information). The soluble fraction of PM was prepared by filtration of the sonicated PM suspension through a Teflon filter (0.45 μm pore size). Metals in the PM suspension and the soluble PM solution were chelated by adding 5 mM of diethylenetriaminepentaacetic acid (DETAPAC).

The reaction started by adding 100 μM of ascorbic acid to different PM solutions containing sufficient amounts of molecular probes. The total volume of each reaction system is 200 μL. Similarly, the reaction was conducted in the 96-well microplate within the microplate reader in darkness at 37 °C. The reaction products of the molecular probes were measured every 15 min over a 2-h period.

The NIST 1648a target concentration used in this study was 250 μg/mL based on ambient PM concentration in US (see Table S3 in the Supplementary Information) and the same dosimetry assumptions described above were made here. The saturation concentration of molecular probe used in this experiment was determined the same way as described above by monitoring the change in fluorescence intensity *vs.* the added amount of molecular probes. Inhibitory controls were also applied by adding 50 mM of DMSO into the reaction system to remove the formed •OH.

## 3. Results

### 3.1. Limit of Detection (LOD)

Calibration curves were generated for each of the reaction products, *i.e.*, 2OHTA, FL, 7OHCCA and 2OHBA in PBS with 100 μM of ascorbic acid (Figure S3 in the Supplementary Information). Based on calibration curves, LOD was calculated as three times the standard deviation of 7 blank samples, which were identical to real samples but without PM or metal ions. The summary of calibration curves and LODs was presented in Table S4 in the Supplementary Information. The LOD of 2OHTA, FL, 7OHCCA and 2OHBA were 17.59, 0.1851, 2.723 and 58.16 nM, respectively.

### 3.2. Reactivity

The reactivity of a molecular probe, which reflects how fast it reacts with •OH, is quantified with a reaction rate constant. A fast reaction is preferred because it indicates a molecular probe could more easily outcompete other substances in the solution to capture •OH [56]. The reaction rate constant has been reported for TPT, 3CCA, and BA [34,39,57,58], but it is unknown for APF. The reaction rate constant of APF was estimated in our study, using the measured reaction rate of APF, the known reaction rate constant of a reference molecular probe (TPT in our case), and the initial concentration of APF added into the reaction system. The estimation is based on a chemical kinetics approach documented by Zhou and Mopper [59].

Briefly, at steady state, the •OH generation rate equals the •OH consumption rate by a molecular probe and all the other unknown scavengers in the reaction system [59]. First order reaction kinetics applies, and the reaction rate constant of •OH consumption by a molecular probe can be expressed as:

(1) $$\frac{1}{R_{p}}=\frac{1}{P_{OH}}+\frac{k_{n}^{\prime }}{P_{OH}k_{p}}\times \frac{1}{\left[Probe\right]}$$

where, *R_p_* is the measured reaction rate between •OH and a molecular probe, *P_OH_* is the steady state •OH generation rate, *k_p_* is the rate constant of the reaction between a molecular probe and •OH, *k*’*_n_* is the rate constant between •OH and all the other scavengers in the reaction system, and [*Probe*] is the molecular probe concentration initially added into the reaction system.

**Figure 1:**
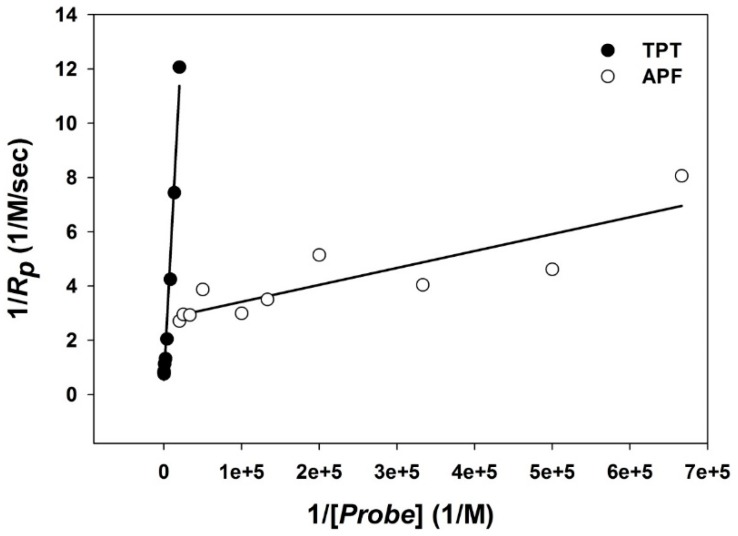
The association of the inverse concentration of molecular probes with inverse reaction rate. Regression equation for TPT is 1/*R_p_* = (5.436 × 10^−4^)/[*TPT*] + 0.5072, *R*^2^ = 0.986 and APF is 1/*R_p_* = (6.243 × 10^−6^)/[*APF*] + 2.793, *R*^2^ = 0.757.

In this study, •OH was induced by Cu^2+^ (see Section 2.3.2. “Hydroxyl Radical Induced by Transition Metal Ions”), and TPT was used as a reference molecular probe, with a known reaction rate constant of *k_p_* = 3.3 × 10^9^ M^−1^s^−1^, to estimate the unknown reaction rate constant of APF. First, the reaction rate between •OH and TPT, expressed as the formation rate of 2OHTA (*R_P_*), was measured for varying amounts of TPT ([*TPT*]). When 1/*R_P_* was regressed on 1/[*TPT*] (Figure 1), *k*’*_n_* was estimated from the slope and the intercept of the regression (Equation 1). The estimated *k*’*_n_* was 9.4 × 10^5^ ± 1.1 × 10^5^ M^−1^s^−1^. Then, APF was applied as the molecular probe under the same experimental settings, and *R_P_* was measured again, as the formation rate of FL, for varying amounts of APF ([*APF*]). When 1/*R_P_* was regressed on 1/[*APF*] (Figure 1), *k_P_* for APF was estimated from the slope and the intercept of the regression, and the estimated *k*’*_n_* from the TPT experiments (Equation 1). The validity of this method was demonstrated by comparing the estimated reaction rate constants of 3CCA and BA, using TPT as a reference molecular probe, and the reaction rate constants of 3CCA and BA reported in the literature [34,39,58,60].

Table 1 presents the estimated reaction rate constants for APF, 3CCA and BA, along with the reaction rate constants of TPT, 3CCA, and BA from the literature. APF has been commercially available only for a short period of time, and the reaction rate constant of APF has been unknown. For the first time, the reaction rate constant was estimated to be 2.9 × 10^11^ M^−1^s^−1^ for APF reacting with •OH.

**Table 1:** Reaction rate constant of molecular probes.

*k_p_* (1/M/s)	TPT	APF	3CCA	BA
Measured in our study	-	2.9 × 10^11^ ± 8.5 × 10^10^	3.4 × 10^9^ ± 2.2 × 10^6^	3.5 × 10^9^ ± 2.3 × 10^8^
Reported in the literature	^a^ 3.3 × 10^9^	-	^b^ 5.01 × 10^9^	^c^ 3.0 × 10^9^

^a^ Barreto *et al*. [60]; ^a^ Mark *et al*. [39]; ^b^ Manevich *et al.* [34]; ^c^ Oturan and Pinson, [58].

Based on the reported reaction rate constant of TPT (3.3 × 10^9^ M^−1^s^−1^) [39,60], the reaction rate constants of 3CCA and BA were also estimated. The estimated and reported reaction rate constants were 3.4 × 10^9^ M^−1^s^−1^ and 5.0 × 10^9^ M^−1^s^−1^, respectively, for 3CCA [34], and 3.5 × 10^9^ M^−1^s^−1^ and 3.0 × 10^9^ M^−1^s^−1^, respectively for BA [58].

### 3.3. Reaction Yield

Reaction yield determines the amount of the fluorescence product formed by the reaction between •OH and each molecular probe. Reaction yield is determined by all the competing stochastic reactions between a molecular probe and •OH. The reaction yields of 2OHTA, 7OHCCA and 2OHBA were reported in previous studies [33,34,56,58,61], but the reaction yield of FL has still been unknown.

Using the reaction yield of forming 2OHTA from TPT and •OH as a reference value, the unknown reaction yield of FL was estimated. A 35% yield of 2OHTA was reported in a reaction between TPT and •OH [56,61]. Based on this reported reaction yield and 2OHTA concentration [2OHTA] measured in our experiments (•OH induced by Cu^2+^), the total amount of produced •OH, which would be captured by a molecular probe, was estimated as [2OHTA]/35%. The reaction yield of FL was estimated as the fraction of the detected FL and the total amount of produced •OH estimated above. The estimated reaction yield of FL formation from APF and •OH was 1%.

The validity of this approach was assessed by comparing the estimated yields from our experiments and the reported yields for 7OHCCA and 2OHBA. The estimated yield of 7OHCCA (the product of 3CCA) and 2OHBA (the product of BA) was 11% and 35%, respectively, compared with reported values of 5.1%–8.8% for 7OHCCA [33,34], and 30% for 2OHBA [58]. Differences between estimated yield and reported value could be attributed to differences in the reaction environments: previous studies used gamma irradiation [33,34] or electrolysis of O_2_ [58] to generate •OH, and lower concentrations of molecular probes (5 mM for 3CCA and 6 mM for BA) were used [33,34,58].

### 3.4. PM-Induced •OH Formation

**Figure 2:**
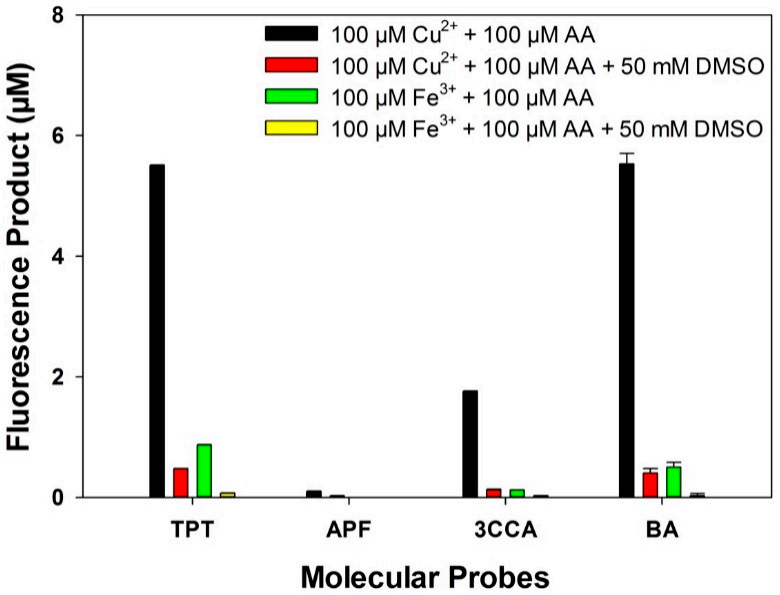
Concentrations of fluorescent products measuring •OH formation induced by Cu^2+^ and Fe^3+^, in PBS with 100 μM ascorbic acid, incubated at 37 °C for 2 h avoiding light.

Figure 2 illustrates the capability of measuring •OH induced by Cu^2+^ or Fe^3+^ for each of the four molecular probes, *i.e.*, TPT, APF, 3CCA, and BA. Under the same condition of •OH generation, the four molecular probes showed differential capability of capturing •OH. Under the condition of 100 μM Cu^2+^ and 100 μM ascorbic acid, all molecular probes detected •OH generation: TPT, APF, 3CCA and BA formed 5.513 μM of 2OHTA, 0.101 μM of FL, 1.754 μM of 7OHCCA, and 5.530 μM of 2OHBA, respectively. Under the condition of 100 μM Fe^3+^ and 100 μM ascorbic acid, APF was unable to detect •OH generation. In contrast, TPT produced 0.872 μM of 2OHTA, 3CCA produced 0.125 μM of 7OHCCA, and BA generated 0.502 μM of 2OHBA. In addition, as the inhibitory control, adding DMSO (50 mM) into the reaction system significantly reduced fluorescence intensity, indicating what the molecular probes captured was indeed •OH. Figure S4 (Supplementary Information) illustrates the time-dependent •OH formation.

**Figure 3:**
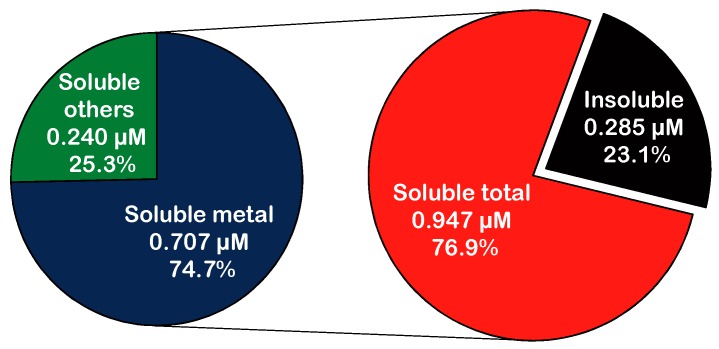
The contribution of different PM fractions on •OH formation. Both percent contributions and the absolute concentrations of 2OHTA are presented. •OH was induced by NIST SRM 1648a in PBS with 100 μM ascorbic acid, incubated at 37 °C for 2 h avoiding light.

Figure 3 illustrates •OH induced by total PM, the soluble PM fraction, and PM with metals removed by DETAPAC. The induced •OH was measured with TPT, which was identified as the most applicable molecular probe among the four molecular probes evaluated (see the Discussion section for details). Total PM (250 μg/mL) and its soluble fraction in PBS with 100 μM ascorbic acid produced 1.232 μM and 0.948 μM of 2OHTA, respectively. DETAPAC chelated with metals and reduced 2OHTA formation from 1.232 μM to 0.469 μM for total PM, and from 0.948 μM to 0.240 μM for the soluble PM fraction. Without ascorbic acid in PBS, both total PM and its soluble fraction produced a negligible amount of •OH (below detection limits). DMSO could also significantly reduce the measured fluorescence intensity, indicating the inhibition of 2OHTA formation by quenching •OH.

## 4. Discussion

### 4.1. Molecular Probe Selection for Ambient PM-Induced •OH

In this study, initial assessments were conducted to evaluate four existing molecular probes for their applicability to measure •OH induced by ambient PM. The evaluations were based on experiments and information in the peer-reviewed literature on assessing the physicochemical properties of the four molecular probes [17,34,56,58]. Based on the six aspects evaluated below, disodium terephthalate (TPT) is recommended as the most applicable molecular probe which can be used in a high-throughput approach to measure •OH induced by ambient PM.

#### 4.1.1. Limit of Detection (LOD)

LODs were measured for each molecular probe in this study. The most sensitive molecular probe was APF (LOD = 0.1851 nM for FL), followed by 3CCA (LOD = 2.723 nM for 7OHCCA), TPT (LOD = 17.59 nM for 2OHTA), and BA (LOD = 58.16 nM for 2OHBA).

#### 4.1.2. Reactivity

The reactivity of a molecular probe, quantified as the reaction rate constant, reflects how fast a molecular probe reacts with •OH. The reaction rate constants of TPT (3.3 × 10^9^ M^−1^s^−1^), 3CCA (5.0 × 10^9^ M^−1^s^−1^), and BA (3.0 × 10^9^ M^−1^s^−1^) were reported in previous studies [34,39,58,60], and the reaction rate constant for APF (2.9 × 10^11^ M^−1^s^−1^) was estimated in this study. The approach to reaction rate constant estimation was reasonable, as indicated by the consistency of the reported rate constant value (see above) and the estimated value for both 3CCA (3.4 × 10^9^ M^−1^s^−1^) and BA (3.5 × 10^9^ M^−1^s^−1^). The reaction rate constants for TPT, 3CCA and BA are on a similar range, and the reaction rate of •OH with APF is about 100 times faster than with other molecular probes.

#### 4.1.3. Reaction Yield

Reaction yield reflects the amount of a product formed and available for fluorescence detection in our study. The reaction yields for 2OHTA (35%), 2OHBA (30%), and 7OHCCA (5.1%~8.8%) were reported in previous studies [33,34,56,58,61], and the yield for FL (1%) was estimated in our study. The estimation approach was confirmed by comparing the estimated and reported yield for 2OHBA and 7OHCCA.

Although both 2OHTA and 2OHBA have the highest reaction yield of about 30%–35% among the four molecular probes evaluated, the yield of hydroxylated derivatives of BA is not constant. The reaction yield of 2OHBA is about 30% at the beginning of reaction, but decreases to 19% at the end due to the increasing amount of tetra- and pentahydroxylated compounds [58]. As a result, 2OHTA has the highest reaction yield among the four molecular probes, and the reaction yield of FL is the lowest.

#### 4.1.4. Product Stability

A stable reaction product is preferred for •OH detection. Fluorescein (FL), which is the product of the •OH reaction with APF, is prone to be further oxidized by •OH forming a non-fluorescent product [17]. Actually, this reaction between FL and •OH has been used in fluorometric assays to quench •OH [62].

Sodium salicylate (2OHBA), a product of the •OH reaction with BA, can continue to react with •OH and produce 2,3-dHBA and 2,5-dHBA. Oturan and Pinson [58] reported that the reaction rate constant of 2OHBA formation was 3.0 × 10^9^ M^−1^s^−1^, while the reaction rate constant of 2OHBA to form 2,3-dHBA and 2,5-dHBA were 1.8 × 10^10^ M^−1^s^−1^ and 0.5 × 10^10^ M^−1^s^−1^, respectively. The formation rate of 2OHBA is slower than the depletion rate of 2OHBA. Indeed, 2OHBA was used as a molecular probe to detect •OH in sulfate-induced colitis and photolysis reaction [36]. The depletion of 2OHBA over time will decrease the measurement accuracy and sensitivity.

7-Hydroxycoumarin-3-carboxylic acid (7OHCCA) is one of the two hydroxylation products of 3CCA, and 7OHCCA is the only product with high fluorescence [34,63]. Manevich *et al.* [34] reported that a small decay (5%) of 7OHCCA fluorescence after incubation of 7OHCCA with the mixture of Cu^2+^ and ascorbic acid, suggesting the stability of 7OHCCA.

TPT has two carboxylate anion side groups attached at position 1 and position 4 of the six-carbon ring to form a structurally symmetrical compound. The reaction between TPT and •OH at any one of the four unsaturated carbons forms only one mono-hydroxylated product, 2OHTA [60]. 2OHTA is stable in the reaction environment, and Saran and Summer [32] reported a less than 3% depletion of 2OHTA within 36 h in the reaction system.

In summary, the products of TPT and 3CCA are most stable among the four fluorescent probes.

#### 4.1.5. The Solubility of a Molecular Probe

The solubility of a fluorescence probe determines its availability in a solution to capture •OH and outcompete other •OH scavengers. The solubility of 3CCA in water is 30 mM, which is low. In our study, the maximum concentration of 3CCA, which can be applied in the reaction system, was 20 mM, and above which precipitation was observed. The low solubility of 3CCA limits its application to measure high concentrations of •OH formation. For APF, 5 mM is the maximum concentration commercially available. In our study, the sufficient amount of APF could not be determined due to this limitation. In contrast, the sodium form of TPT and BA has high solubility in water, 0.7 M for TPT [64] and 3.8 M for BA [65], and can be used to measure high rates of •OH formation.

#### 4.1.6. Fluorescence Intensity Affected by pH

The fluorescence intensity of the fluorescent products is affected by the pH of the reaction environment. The fluorescence intensity of 2OHTA is constant between pH 6 to 11, but a sharp decrease occurs below pH = 4 and shows much less fluorescence intensity (10%) at pH = 2 [57]. Fluorescence intensity of 2OHBA is also stable between pH 6 to 11 and has similar decrement with 2OHTA [57]. 7OHCCA is highly fluorescent, but the fluorescence intensity of 7OHCCA is stable when pH > 9.0 and decreases over 70% at pH 7.0 [34]. Cohn *et al.* [24] reported that the fluorescence intensity of FL increased as the pH increased, and a sharp increase was observed when pH > 11. A stable fluorescence intensity around pH = 7 is an ideal property in our study, which could minimize the impact of pH on measurement accuracy. In summary, 2OHTA and 2OHBA have stable fluorescence under the physiologically relevant condition (pH 7.4), but 7OHCCA and FL have high fluorescence intensity in basic conditions (pH > 9.0).

#### 4.1.7. A Brief Summary of the Molecular Probes Evaluated in the Study

The physicochemical properties evaluated across different molecular probes are summarized in Table 2. Among the four molecular probes evaluated, there is no one molecular probe superior to others in all six aspects evaluated.

**Table 2:** Summary of molecular probe properties.

Molecular Probe Properties	Molecular Probe			
	TPT	APF	3CCA	BA
LOD (nM)	17.59	0.1851	2.723	58.16
Reactivity (1/M/s)	3.3 × 10^9 a^	2.9 × 10^11^	3.4 × 10^9^	3.5 × 10^9^
Yield (%)	35 ^b^	1	11	35
Product stability	High	Low	High	Low
Solubility	High	-	Low	High
Optimal pH range	6~11	>9	>9	6~11

^a^ Barreto *et al.* [60] and Mark *et al.* [39]; ^b^ Fang *et al.* [61] and Page *et al.* [56].

However, TPT is considered as the most applicable molecular probe to measure •OH induced by PM using a high-throughput format, due to its high solubility, high stability of the corresponding fluorescent product, high yield compared with others, and stable fluorescence intensity in a wide range of pH environment. The application of APF is limited by its solubility, the optimal pH range for fluorescent detection, low yield, and low stability of the fluorescent product. Factors limiting the applications of 3CCA include its solubility, the optimal pH range for fluorescent detection. For BA, the limiting factor is its low fluorescent product stability and specificity.

### 4.2. Hydroxyl Radicals Induced by Ambient PM

Oxidative potential of PM has been used to characterize the ability of PM to cause oxidative stress [10], which has been identified as one of the key mechanisms linking ambient PM exposure with adverse health effects [1]. Although efforts have been made to quantify •OH, the most destructive ROS, no high-throughput methods have been available to date to specifically measure •OH induced by ambient PM, presumably due to the lack of a reliable molecular probe. In this study, we evaluated four molecular probes and identified TPT as the most applicable and promising molecular probe for •OH measurement.

TPT was tested using a standard reference material, and was applied to measure •OH induced by PM and its fractions using a high-throughput format. TPT was able to detect •OH formation under physiologically relevant conditions relevant to real-world PM exposure levels. TPT was also able to measure •OH induced by both total PM, the soluble fraction of PM, and PM with metals inhibited by DETAPAC, which can slow down the Fenton reaction [42,66]. The soluble fraction contributed approximately 76.9% of the •OH induced by PM, and the soluble metals ions of PM contributed 57.4% of the overall •OH formation. Soluble metal ions were major contributors to •OH formation, which is consistent with previous findings. Both water soluble Cu^2+^ and Fe^3+^ were highly correlated with ROS formation, induced by PM_2.5_ and PM_10_ [43,67,68]. Hu *et al.* [69] reported that water soluble elements in coarse, accumulation and quasi-ultrafine particles were associated with both increased macrophage ROS levels and increased consumption of DTT in a cell free system. ROS activity, which was measured using DCFH-DA in rat alveoli macrophage was also significantly associated with water soluble transition metal ions found in PM_2.5_ [70,71], PM_10_ [70] and coarse particles [72].

It is also worth noting that both total PM and the soluble fraction of PM induced dramatically lower •OH when ascorbic acid was not present, indicating that ascorbic acid contributed to the redox cycle of transition metals and enhanced Fenton reaction [41].

In future measurements of PM-induced •OH, TPT could be applied in an automated system (e.g., a particle-into-liquid sampler) to achieve continuous and high-throughput measurements. We expect the TPT concentration used in our study could be applied in most future studies to measure ambient PM induced •OH, because our study reflects real-world high pollution scenarios in the US. However, if a study is conducted in a highly polluted area outside the US, the TPT concentration needs to be re-estimated based on saturation experiments described above.

### 4.3. Strengths and Limitations

This study reflects our initial efforts to find a suitable molecular probe, which is potentially capable to be applied in a high-throughput approach, to measure •OH induced by ambient PM. The following limitations or further research needs are identified: (1) The molecular probes were tested using a NIST Standard Reference PM, not ambient PM. (2) •OH measured using this approach reflects the intrinsic redox potential only in an acellular model. Further study is needed to examine the associations of this assay and intracellular oxidative stress, although previous studies have demonstrated the associations of PM oxidative potential and intracellular oxidative stress [3]. (3) Ascorbic acid is one of the antioxidants in epithelial lining fluids and in mammalian cells. In future studies, a more complex assay will be developed to account for the contributions from other antioxidants (e.g., glutathione and uric acid) to •OH formation. Previous studies have used ascorbic acid only assay to measure PM oxidative potential and indicated that ascorbic acid plays the major role in ROS formation, compared with other antioxidants [23].

Despite these limitations, a systematic evaluation has been conducted to find a suitable molecular probe for measuring •OH induced by ambient PM which can be used in a high-throughput approach. The molecular probe we identified was successfully applied to measure •OH induced by different fractions of PM. This effort can lead to a routine and cost-effective measurement (see Table S1 in the Supplementary Information) of •OH induced by PM in future exposure and health impact studies.

## 5. Conclusions

Four existing molecular probes (TPT, APF, 3CCA and BA) were evaluated, in terms of their sensitivity (LOD), their reactivity with •OH, the yield of the corresponding fluorescent products, the stability of the fluorescent products, their solubility, and the impact of pH on a product’s fluorescence intensity. Among the four molecular probes, TPT was judged as the most applicable molecular probe, due to its high solubility, high stability of the corresponding fluorescent product, high yield compared with others, and stable fluorescence intensity in a wide range of pH environment. We also, for the first time, report the APF reaction rate constant with •OH (2.9 × 10^11^ M^−1^s^−1^) and the reaction yield (1%). TPT detected •OH formation induced by total PM and the soluble fraction of PM. The soluble fraction contributed approximately 76.9% of the overall •OH formation, and the soluble metals ions contributed 57.4% of the overall •OH formation. This study provides a promising high-throughput method to measure •OH induced by PM on a routine basis.

## Supplementary Files

Supplementary File 1
