# Supplementary material for: A Novel High-Throughput Approach to Measure Hydroxyl Radicals Induced by Airborne Particulate Matter
Source: Int J Environ Res Public Health. 2015 Oct 28;12(11):13678–95. doi: 10.3390/ijerph121113678 (PMC4661607; doi:10.3390/ijerph121113678)
Supplement: Supplementary File 1 [file ijerph-12-13678-s001.pdf]

A Novel High-Throughput Approach to Measure Hydroxyl Radicals Induced by Airborne Particulate Matter

1. Comparison of Selected ROS Measurement Methods

Table S1. Advantages, limitations and analytical cost of selected ROS measurement methods.

Methods	Target ROS	Advantages	Limitations	Analytical Cost
Antioxidant depletion [a] (e.g., ascorbic acid)	Nonspecific	(1) Reflects reactions likely to occur *in vivo* at the air-lung interface	(1) Antioxidants, especially ascorbic acid, can react with PM constituents, e.g., transition metal ion or quinones (2) ROS nonspecific measure	(1) Ascorbic acid (39$/25 g, Sigma A5960), HPLC solvent, column (2) HPLC/electro-chemical detection; 1 sample take at least 10 min
DTT assay [b]	O_2^-, H_2O_2, •OH	(1) Simulate *in vivo* electron-transfer mechanisms (2) Surrogate measure of the *in vivo* ROS formation capacity induced by PM	(1) Various PM components participate in DTT activity; e.g., PAHs, quinones, transition metals (2) ROS nonspecific measure	(1) DTT (211.5$/10 g, Sigma D0632) (2) Routine system can measure a sample per 1 h
ESR [c]	Depends on spin trap/probe and/or inhibitor	(1) Direct ROS measurement (2) High sensitivity (3) Can be applied to cellular oxidative stress measurements	(1) Result can be altered by ferric ion in PM (2) Short lifetime of spin trap/probe adduct	(1) DMPO (90.2$/100 mg, Sigma 92688) (2) Cost of ESR can be expensive than other spectrometric methods
DCFH-DA [d]	H_2O_2, •OH, •ROO, •NO, $ONOO^-$	(1) Good marker for the cellular oxidative stress	(1) DCFH react with Horseradish peroxidase, xanthine oxidase or SOD (2) High background signal (3) Auto-oxidation of product (DCF)	(1) DCFH-DA(138.5$/250 mg) (2) HPLC may needs more maintenance costs then fluorescence spectrometer
Sodium benzoate [d]	•OH	See main text	See main text	(1) Sodium benzoate (50.7$/250 g, Sigma B3420)
3CCA [d]	•OH	See main text	See main text	(1) 3CCA (56.1$/25 g, Sigma C85603)
APF [d]	•OH, HOCl	See main text	See main text	(1) APF (459$/1 mg, Sigma A4108)
TPT [d]	•OH	See main text	See main text	(1) TPT (86.1 g/100 g, Alfa Aesar 42946) (2) 96 sample per a measurement using fluorescence spectrometer in a minute

[a] Ayres *et al.* [1]; [b] Fang *et al.* [2]; [c] Khan *et al.* [3]; Makino *et al.* [4]; [d] Gomes *et al.* [5].

2. Chemical Purity and Manufactures

Table S2. The purities and manufacturers of chemicals.

Abbreviation	Name	Manufacturer
TPT	Disodium terephthalate (≥99.0%)	Alfa Aesar
APF	3′-*p*-(Aminophenyl) fluorescein (5 mM solution in dimethylformamide)	Molecular Probes
3CCA	Coumarin-3-carboxylic acid (99%)	Sigma-Aldrich
BA	Sodium benzoate (≥99.0%)	Sigma-Aldrich
2OHTA	2-Hydroxyterephthalic acid (97%)	Sigma-Aldrich
FL	Fluorescein sodium salt (≥95.0%)	Fluka
7OHCCA	7-Hydroxycoumarin-3-carboxylic acid (≥98.0%)	Sigma-Aldrich
2OHBA	Sodium salicylate (≥99.5%)	Sigma-Aldrich
DMSO	Dimethyl sulfoxide (≥99.9%)	Sigma-Aldrich
DETAPAC	Diethylenetriaminepentaacetic acid (≥99%)	Sigma-Aldrich
AA	L-ascorbic acid (≥99.0%)	Sigma-Aldrich
	Sodium chloride (≥99.5%)	Sigma-Aldrich
	Sodium hydroxide (≥98%)	Sigma-Aldrich
	Sodium phosphate dibasic (≥99.0%)	Sigma-Aldrich
	Potassium phosphate monobasic (≥99.995%)	Fluka
	10 mg/mL of single Fe^{3+} and Cu^{2+} ion solution in 10% HNO_3	High-Purity Standards
	Standard urban PM sample (SRM 1648a)	NIST
	Sodium form of Chelex-100 resin (50–100 mesh)	Sigma-Aldrich

3. Finding Sufficient Amount of Molecular Probes in the Experiment

Figure S1 represents saturation experiment results using Cu^{2+} and Fe^{3+} with each fluorescence probe. The saturation experiment was conducted using different concentrations of fluorescence probes with 100 µM of Cu^{2+} or Fe^{3+} in PBS containing 100 µM of ascorbic acid. Fluorescence probe stock solutions, TPT, 3CCA and BA, were prepared and stored in −20 °C, avoid light. 200 mM of TPT and BA was dissolved in PBS. 20 mM of 3CCA was prepared using 50 mM Na_2HPO_4, pH 9.0. 3CCA stock solution was incubated overnight at 60 °C. The pH of the final 3CCA stock solution was adjusted to 7.4 using 50% sodium hydroxide solution. The purchased of 5mM APF solution was directly used to detect •OH. The saturation or plateau was observed after adding sufficient concentration of fluorescence probe, which can compete with unknown scavengers. The plateau region was observed after adding 2.5 mM of TPT and BA, or 7.5 mM of 3CCA with Fe^{3+} and 2.5 mM with Cu^{2+}. APF did not shown clear plateau with Fe^{3+} because its autoxidation rate was faster than the fluorescence increase rate. Consequently, the final concentration of each fluorescence probes in the subsequent experiments was determined based on Figure S1: 10 mM for TPT, 50 µM for APF, 10 mM for BA and 15 mM for 3CCA.

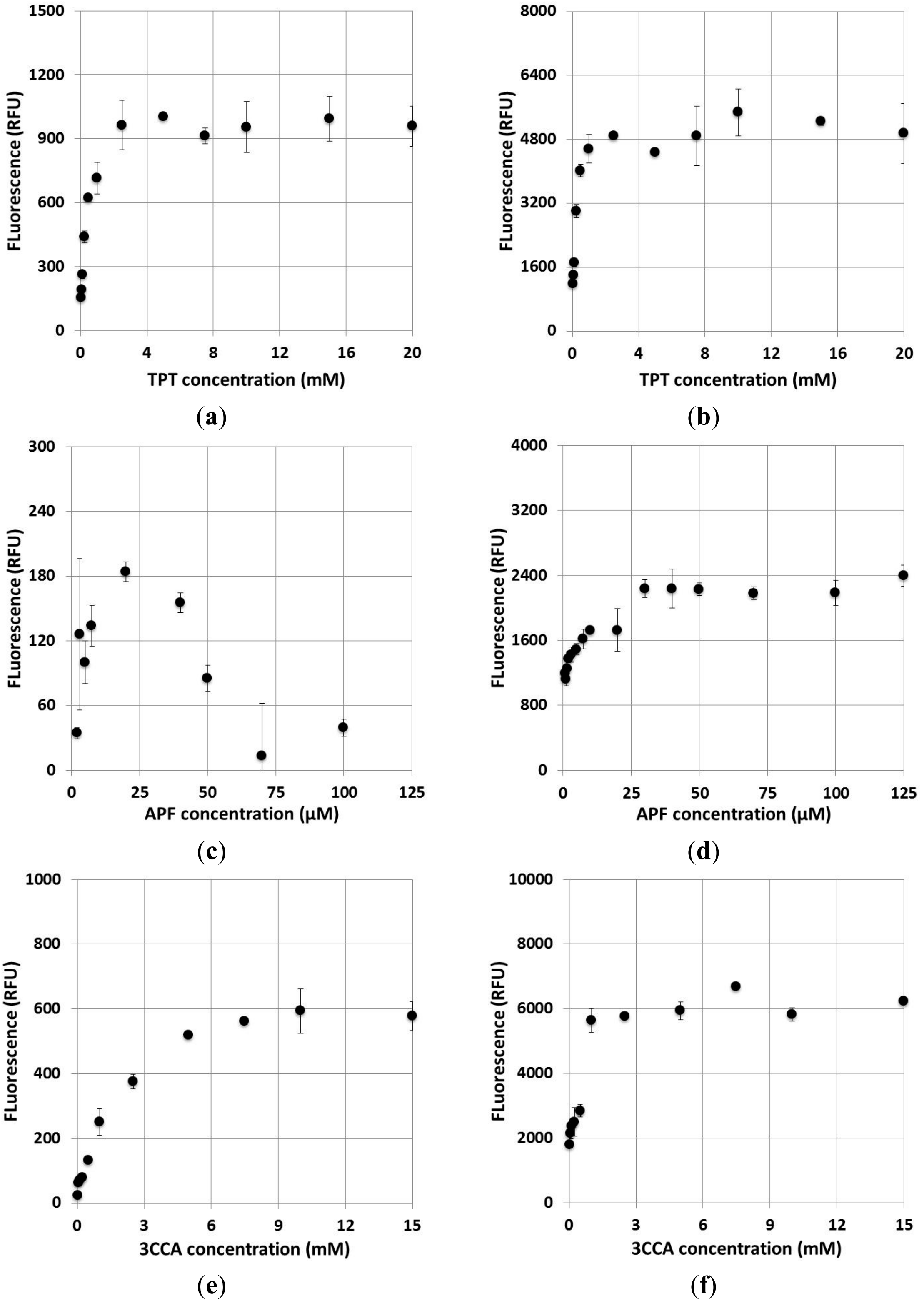

Figure S1. *Cont.*

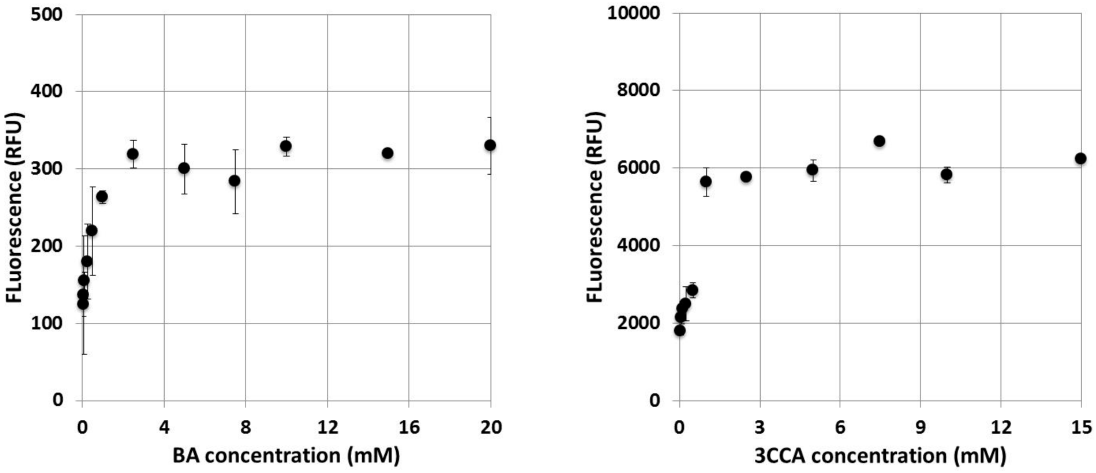

(g) (h)

Figure S1. Fluorescence intensity using different concentration of fluorescence probes, TPT, APF, 3CCA and BA, with 100 µM Cu^{2+} and 100 µM AA and 100 µM Fe^{3+} and 100 µM AA in SBF, pH 7.4 after 2 h incubation at 37 °C in the dark. (**a**) 100 µM Fe^{3+} + 100 µM AA + TPT; (**b**) 100 µM Cu^{2+} + 100 µM AA + TPT; (**c**) 100 µM Fe^{3+} + 100 µM AA + APF; (**d**) 100 µM Cu^{2+} + 100 µM AA + APF; (**e**) 100 µM Fe^{3+} + 100 µM AA + 3CCA; (**f**) 100 µM Cu^{2+} + 100 µM AA + 3CCA; (**g**) 100 µM Fe^{3+} + 100 µM AA + BA; (**h**) 100 µM Cu^{2+} + 100 µM AA + BA.

4. Ambient PM Concentrations and Its Constituents in the US

Table S3. Ambient PM (µg/m^3) and transition metal (ng/m^3) concentrations in the US.

$PM_{2.5}$ (µg/m^3)/Metal (ng/m^3)	Concentration											
	**Mean**	**Min**	**P1**	**P5**	**P10**	**P25**	**Median**	**P75**	**P90**	**P95**	**P99**	**Max**
$PM_{2.5}$	9.2	1.0	5.4	7.7	9.3	15.0	20.4	25.7	33.1	39.4	71.9	236.2
Cd	1.4	−0.4	0.0	0.0	0.0	0.0	0.0	0.5	5.1	8.6	16.7	46.4
Cr	2.5	−0.5	0.0	0.0	0.0	0.0	0.4	1.8	4.2	7.1	27.4	1580.0
Cu	5.0	−0.5	0.0	0.0	0.2	1.0	2.5	5.3	10.7	16.7	41.0	1270.0
Fe	98.4	0.0	5.7	14.0	20.5	35.9	63.1	112.0	194.0	283.0	615.0	10,400.0
Mn	3.7	0.0	0.0	0.0	0.0	0.5	1.5	3.2	6.2	10.0	28.6	2560.0
Ni	1.7	−0.4	0.0	0.0	0.0	0.1	0.7	1.5	3.5	6.0	18.5	474.0
Pb	4.1	−0.4	0.0	0.0	0.0	0.4	2.2	5.0	9.2	13.2	30.2	980.0
V	2.5	−0.1	0.0	0.0	0.0	0.1	1.3	3.0	6.1	9.1	18.8	182.3
Zn	15.9	0.0	0.0	1.1	2.0	4.3	8.3	15.6	30.0	47.7	131.0	3110.0

The mean $PM_{2.5}$ concentration and 9 commonly observed metals in the air (Cd, Cr, Cu, Fe, Mn, Ni, Pb, V, and Zn) during 2012 (Table S3) was used to calculate the equivalent exposure concentrations based on air concentrations and dosimetry of 24 h exposure. The following assumptions are made during the calculation: (1) The inhalation rate is 20 m^3/day, (2) Percent of particles deposited in the lung is 20%, (3) The volume of epithelial lining fluid is 25 mL, (4) Metal concentrations in the hotspot in the lung are 100 times higher than the average concentrations. The equivalent concentration for Cu, Fe and PM in this study was determined as 100 µM, 100 µM and 250 µg/mL, respectively.

5. PM Sonication Time

Effect of sonication and incubation time was evaluated and presented in Figure S2. 2OHTA concentration for 2, 5, and 10 min sonication and 0 h incubation of TPM was 1.885, 2.337 and 1.878 µM, respectively. 2OHTA concentration for 2, 5, and 10 min sonication and 24 h incubation of total PM was 1.792, 1.562 and 1.702 µM, respectively. 2OHTA concentration range for soluble sample was from 1.020 to 1.742 µM. There was no large difference in 2OHTA formation across sonication and incubation times. Consequently, 5 min of sonication and 0 h incubation was used in PM experiments.

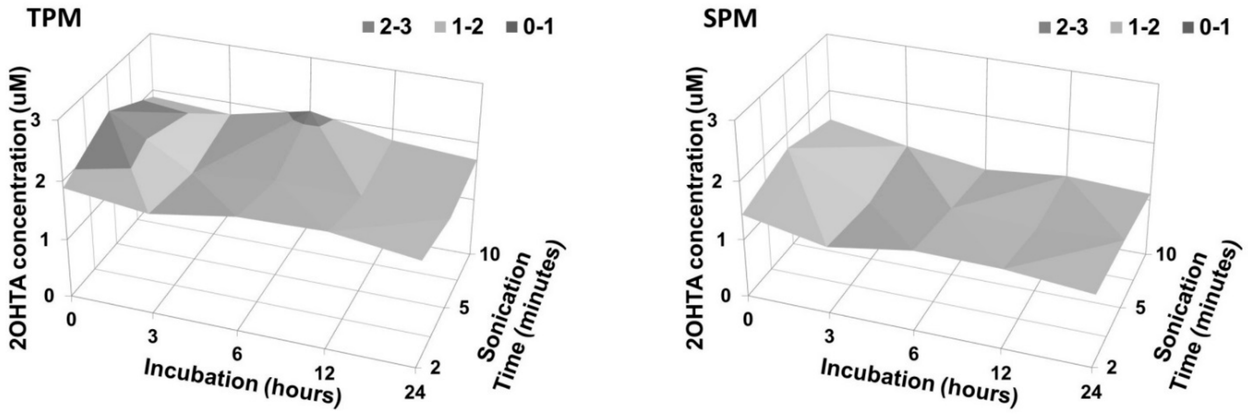

Figure S2. 2OHTA concentration under different sonication and incubation conditions.

6. Calibration Curves of the Florescent Products

Table S4. Summary of calibration curve and limit of detection.

Product	Slope	Intercept	LOD (nM)	R^2
2OHTA	974.71	104.9	17.59	0.998
FL	16638	556.2	0.1851	0.999
7OHCCA	3466.0	144.5	2.723	0.998
2OHBA	404.76	125.8	58.16	0.998

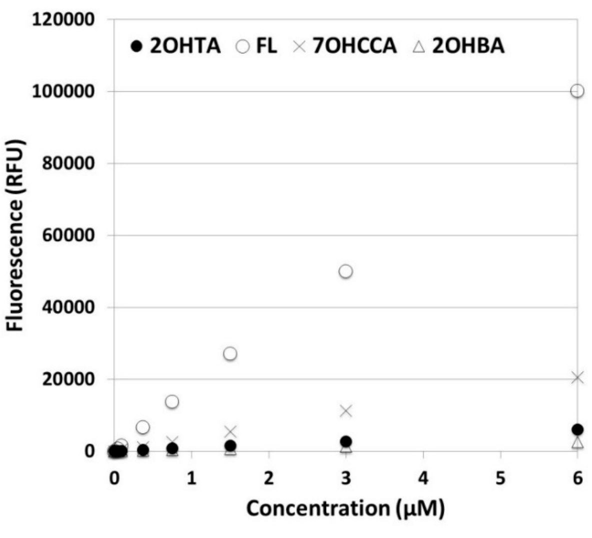

Figure S3. Calibration curves for 2OHTA, FL, 7OHCCA and 2OHBA.

7. The Time-Dependent •OH Formation

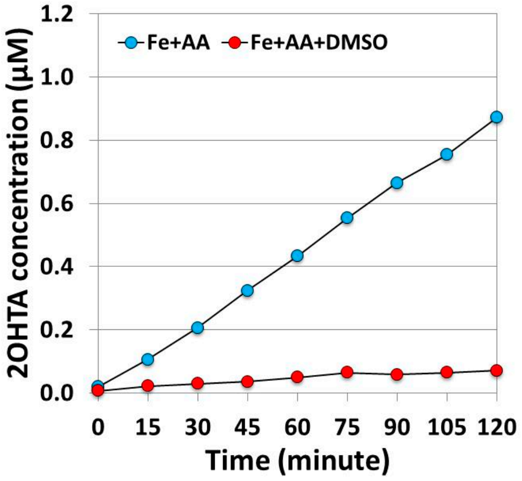

(**a**)

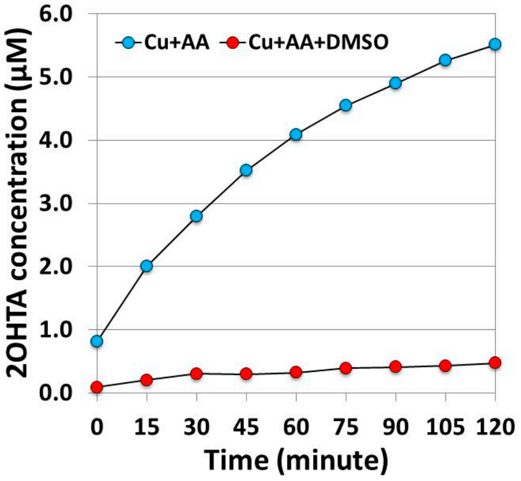

(**b**)

Not available due to insufficient APF concentrations, as discussed in the main text.

(**c**)

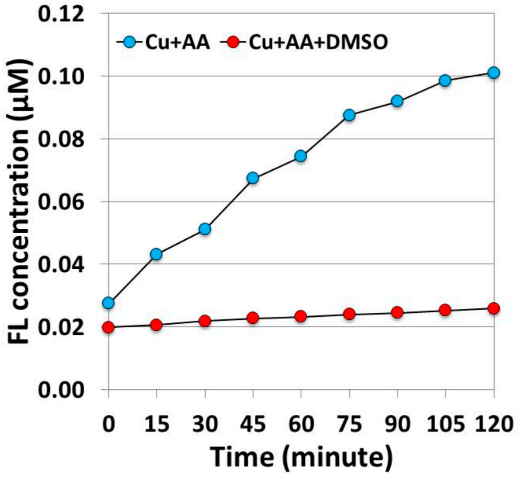

(**d**)

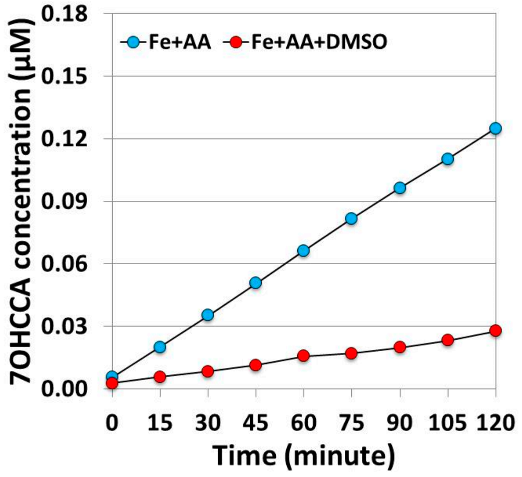

(**e**)

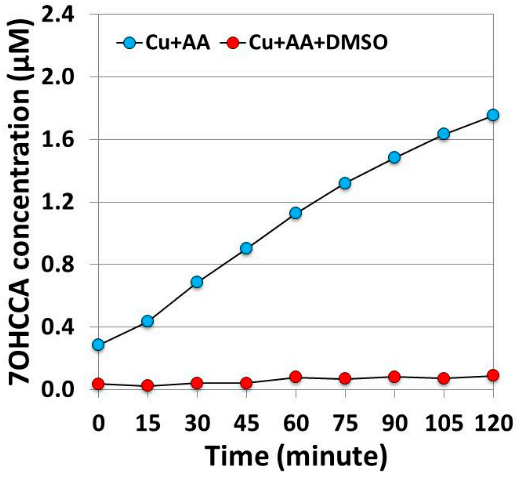

(**f**)

Figure S4. *Cont.*

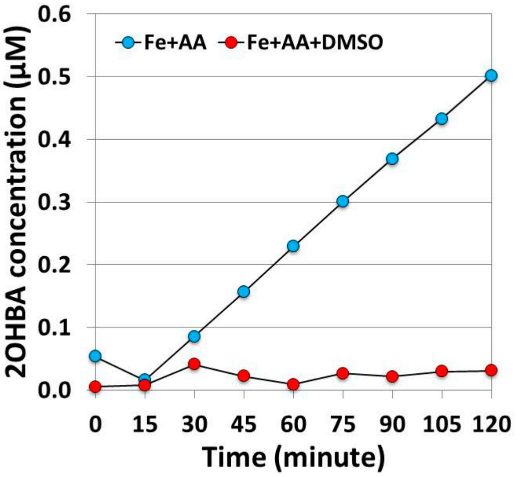

(**g**)

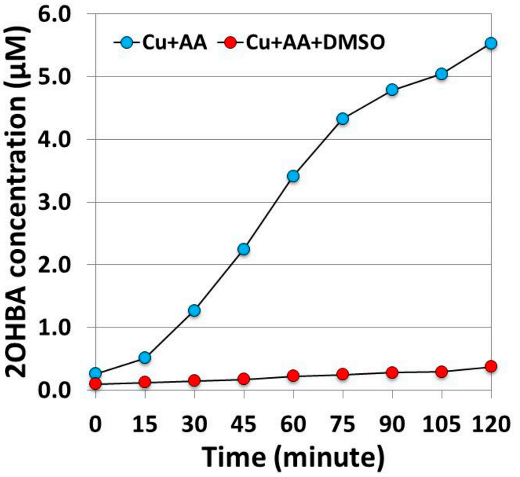

(**h**)

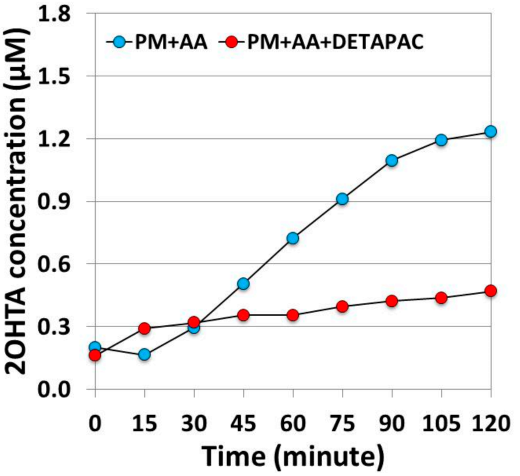

(**i**)

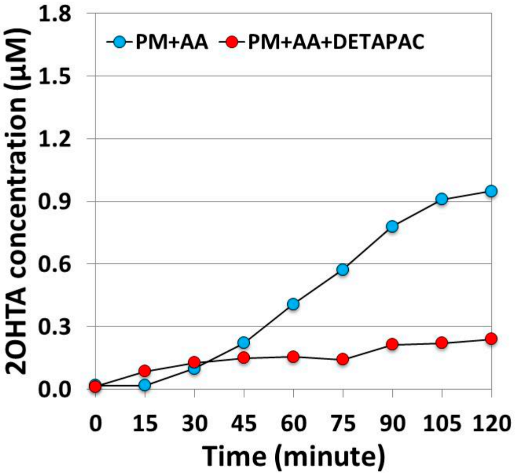

(**j**)

Figure S4. Time-dependent •OH formation, induced by Cu^{2+}, Fe^{3+} and PM in PBS with 100 µM ascorbic acid, incubated at 37 °C. (**a**) 100 µM Fe^{3+} + 100 µM AA + TPT; (**b**) 100 µM Cu^{2+} + 100 µM AA + TPT; (**c**) 100 µM Fe^{3+} + 100 µM AA + APF; (**d**) 100 µM Cu^{2+} + 100 µM AA + APF; (**e**) 100 µM Fe^{3+} + 100 µM AA + 3CCA; (**f**) 100 µM Cu^{2+} + 100 µM AA + 3CCA; (**g**) 100 µM Fe^{3+} + 100 µM AA + BA; (**h**) 100 µM Cu^{2+} + 100 µM AA + BA; (**i**) Total PM + 100 µM AA + TPT; (**j**) Soluble PM + 100 µM AA + TPT.

8. The Contribution of Different PM Fractions on •OH Formation

Table S5. The contribution of PM fractions on •OH formation (measured as 2OHTA formation).

PM Fractions	Rate of 2OHTA Formation (μM/sec)	Concentration of 2OHTA (μM)	Percentage of Formed 2OHTA	
			% Total	% Soluble
Total PM	1.71×10^{-4}	1.232	100	-
Insoluble PM	3.95×10^{-5}	0.285	23.1	-
Soluble PM	1.32×10^{-4}	0.948	76.9	100
Soluble metal	9.83×10^{-5}	0.707	57.4	74.7
Soluble others	3.33×10^{-5}	0.24	19.5	25.3
Total PM + DETAPAC	6.51×10^{-5}	0.469	38.1	-
Soluble PM + DETAPAC	3.33×10^{-5}	0.24	19.5	-

© 2015 by the authors; licensee MDPI, Basel, Switzerland. This article is an open access article distributed under the terms and conditions of the Creative Commons Attribution license (http://creativecommons.org/licenses/by/4.0/).
